# Comparative Analysis of Mitogenomes of *Chironomus* (Diptera: Chironomidae)

**DOI:** 10.3390/insects13121164

**Published:** 2022-12-16

**Authors:** Shu-Yi Li, Yan-Min Zhao, Bing-Xin Guo, Chen-Hong Li, Bing-Jiao Sun, Xiao-Long Lin

**Affiliations:** 1 Engineering Research Center of Environmental DNA and Ecological Water Health Assessment, Shanghai Ocean University, Shanghai 201306, China; 2 Shanghai Universities Key Laboratory of Marine Animal Taxonomy and Evolution, Shanghai Ocean University, Shanghai 201306, China; 3 State Key Laboratory of Environmental Criteria and Risk Assessment, Chinese Research Academy of Environmental Sciences, Beijing 100012, China; 4 China National Environmental Monitoring Centre, Beijing 100012, China

**Keywords:** mitogenome, phylogeny, *Chironomus*

## Abstract

**Simple Summary:**

Mitochondrial genomes (mitogenomes) have been widely used for studying the taxonomy and phylogeny of insects. Chironomids are important bioindicators for monitoring and assessing the health of freshwater ecosystems. However, only a few complete mitogenomes of *Chironomus* species have been reported till now. In this study, the whole mitogenome sequences of 12 *Chironomus* species and one *Microchironomus* species are reported for the first time. Coupled with published mitogenomes, the nucleotide composition, codon usage, PCG selection pressure, and heterogeneity of the mitogenomes of 15 *Chironomus* species were analyzed. The phylogenetic relationships of *Chironomus* based on mitogenomes were reconstructed. The result showed that the mitogenomes of *Chironomus* species were conservative in respect of nucleotide composition and gene order. Our study enriches the library of mitogenomes of chironomids and provides a valuable resource for understanding the evolutionary history of *Chironomus*.

**Abstract:**

(1) Background: Chironomids are biological indicators, playing an important role in monitoring and assessing the changes in water ecosystems. Mitochondrial genomes have been widely applied as a molecular marker to analyze the taxonomy and phylogeny of insects. However, knowledge of the mitogenomes of *Chironomus* species is scarce at present, which limits our understanding of the evolutionary relationships among *Chironomus*. (2) Methods: In our study, the mitogenomes and their basic structure of 12 *Chironomus* species and one *Microchironomus* species were newly sequenced. Combined with reported mitogenomes, a total of 15 mitogenomes of *Chironomus* were selected for a comparative mitogenomic analysis and phylogenetic reconstruction of *Chironomus*. (3) Results: Each mitogenome of the *Chironomus* species has the typical 37 genes and a control region. The basic structure of the whole mitogenomes of *Chironomus* species is relatively conservative, and the genetic arrangements stay the same as the ancestral mitogenome. (4) Conclusions: Our study enriches the library of mitogenomes of chironomids and provides a valuable resource for understanding the evolutionary history of *Chironomus*.

## 1. Introduction

The mitochondrial genome (mitogenome) of insects is a 14–20 kb circular molecule, including 13 protein-coding genes (PCGs), two ribosomal RNAs (rRNAs), 22 transfer RNAs (tRNAs), and one non-coding control region (CR) [1]. Mitogenomes are considered as useful molecular markers for phylogenetic and evolutionary analysis in many insect groups [2,3,4,5] due to their small genome size, maternal inheritance, low sequence recombination, and fast evolutionary rates [6,7]. With the wide application of the high-throughput sequencing technology, mitogenomes have proven successful in species delimitation and phylogenetics among aquatic insects [4,5,8,9,10,11,12,13,14,15].

Chironomidae is one of the most abundant and species-diverse groups of freshwater zoobenthos, containing over 6300 described species worldwide (P. Ashe pers comm.). Chironomid larvae can be found in all types of water bodies and are regarded as significant bioindicators for monitoring and assessing the health of freshwater ecosystems. *Chironomus* is the type genus of Chironomidae and includes over 300 described species distributed over the world except Antarctica [16]. Species delimitation and phylogeny within *Chironomus* have been conducted by morphology or a few genetic markers in previous studies [17,18]. Hence, phylogenetics among *Chironomus* has never been tested based on mitogenomes. Prior to this study, the mitogenomes of three *Chironomus* species have been reported [19,20,21], and the comparative analysis of nucleotide composition and evolutionary rates within the genus have never been carried out.

In this study, we provided new mitogenomes of 12 *Chironomus* species and one *Microchironomus* species. Combined with the reported mitogenomes of three *Chironomus* species, we investigated the basic characteristics of these mitogenomes of *Chironomus*.

## 2. Materials and Methods

### 2.1. Taxon Sampling and Sequencing

The 12 species of *Chironomus* and one species of *Microchironomus* were collected from China, Namibia, New Caledonia and Norway, and used for mitogenome sequencing (Table 1). In addition, mitogenomes of *Chironomus tepperi*, *Chironomus flaviplumus*, *Chironomus kiiensis*, and *Microchironomus tabarui* were retrieved from GenBank for comparative mitogenomic analysis and phylogeny. The vouchers are deposited at College of Fisheries and Life, Shanghai Ocean University, Shanghai, China. The total genomic DNA was extracted from the thorax of an adult or larva using the Qiagen DNA blood and tissue Kit (Qiagen, Hilden, Germany). The genomes of 13 species were sequenced using the Illumina NovaSeq 6000 platform with an insert size of 350 bp and a paired-end 150 bp sequencing strategy at Novogene Co., Ltd. (Beijing, China). The raw reads were trimmed of adapters by Trimmomatic [22], and approximately 3 Gb of clean data in each sample was obtained.

### 2.2. Genome Assembly and Annotation

The seed sequence COI of each species was obtained on GenBank for verification during assembly. The mitogenome sequences were *de novo* assembled using NovoPlasty v 4.2 [24] with 39 kmer and IDBA-UD [25] with the minimum and maximum kmer values of 40 and 120 bp, respectively. In order to check the correctness of the mitogenome sequences, we used Geneious [26] to compare the obtained sequences and compile them into a single sequence. Transport RNA (tRNA) genes were detected on MITOS2 web server (http://mitos2.bioinf.uni-leipzig.de/index.py, accessed on 20 May 2022). The rRNAs and PCGs were annotated manually with the *Chironomus tepperi* as a reference using Clustal Omega in Geneious. Finally, the new mitogenome sequences were deposited in GenBank of NCBI (ON975023–ON975035).

### 2.3. Sequence Analyses

Nucleotide composition of the mitogenome and each type of gene were calculated using SeqKit [27]. The bias of AT and CG were measured according to the formulas: AT-skew = (A − T)/(A + T) and GC-skew = (G − C)/(G + C). Codon family usage of protein-coding gene and relative synonymous codon usage (RSCU) were assessed in MEGA 11 [28]. Non-synonymous substitution rate (Ka) and synonymous substitution rate (Ks) of 13 PCGs were calculated in DnaSP 6 [29].

### 2.4. Phylogenetic Analyses

The mitogenome map was depicted with CGview server [30]. In this study, 12 newly sequenced *Chironomus* species and three *Chironomus* species retrieved on GenBank were selected as the ingroups of phylogenetic analysis, and two species of *Microchironomus* (*Microchironomus tabarui* and *Microchironomus tener*) were selected as the outgroups. Thirteen PCGs and two rRNAs of each species were individually compared using MAFFT [31], and then trimmed using trimAl [32] to align the sequence. FASconCAT-G_v1.05 [33] was used to concatenate aligned sequences of each gene and generate 4 datasets: (1) PCG123 (all codon positions of the 13 PCGs) contained 11,175 sites; (2) PCG123R (all codon positions of the 13 PCGs and two rRNAs) contained 13,357 sites; (3) PCG12R (the 1st and 2nd codon positions of the 13 PCGs and two rRNAs) contained 9632 sites; (4) AA (amino acid sequences of the 13 PCGs) contained 3725 sites. Transition and transversion rates were evaluated in DAMBE to test the level of base substitution saturation [34]. The substitution of each dataset (PCG123R, PCG12R, and PCG123) was not saturated (Figure S1). The heterogeneity analysis of four datasets was performed with AliGROOVE_1.06 [35]. The best partitioning scheme and the best-fit substitution model inferred for each partition were tested using PartitionFinder 2.0 [36] with the Bayesian Information Criterion. Phylogenetic analyses were carried out with Bayesian inference (BI) and maximum likelihood (ML) reconstruction. BI analysis was conducted using MrBayes 3.2.7 [37] with the best-fit substitution model (Table S1). Markov chain Monte Carlo (MCMC) was run twice for 10,000,000 generations, trees were sampled once every 1000 generations, and the first 25% of trees were discarded as burin-in. The chains were stopped after the two runs had satisfactorily converged. The ML analysis was conducted using IQ-TREE 2 [38] with the best-fit substitution model (Table S1) and 1000 bootstrap replicates.

## 3. Results and Discussion

### 3.1. Basic Structure of Chironomus Mitogenomes

**Figure 1:**
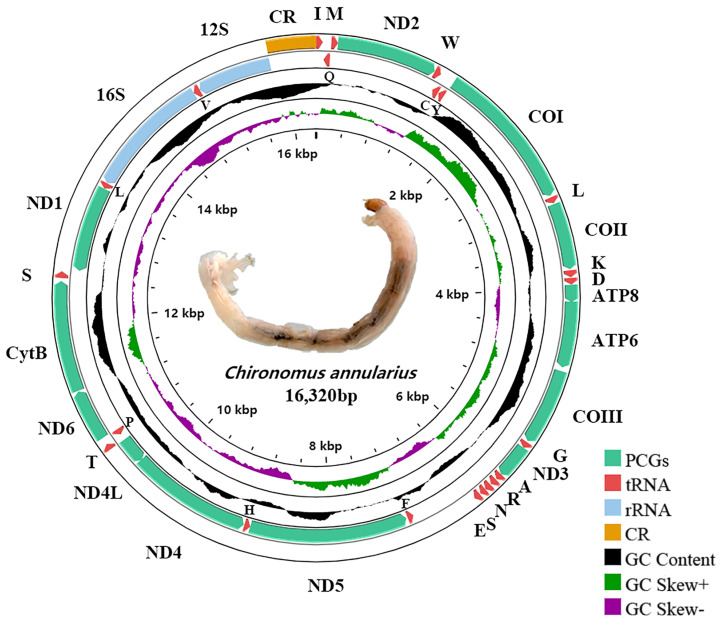
Mitogenome map of representative species of *Chironomus* (*Chironomus annularius*). The arrow indicates the direction of gene transcription. PCGs and rRNAs are represented by normative abbreviations, while tRNAs are represented by single-letter abbreviations. In the notes at the bottom right, green, red, blue, and yellow respectively corresponded to PCGs, tRNAs, rRNAs, and CR. The second circle shows the G+C content of the complete mitogenome. The third circle exhibits the GC-skew of the whole mitogenome. The innermost circle shows the morphology of the larvae of *Chironomus annularius* and the length of the mitogenome.

The length of newly sequenced whole mitogenomes of *C*. *anthracinus*, *C*. *nipponensis*, *C*. *flaviplumus*, *C*. *plumosus*, *C*. *tentans*, *C*. *novosibiricus*, *C*. *annularius*, *C*. *agilis*, *C*. *nippodorsalis*, *C*. *transvaalensis*, *C*. *circumdatus*, and *C*. *javanus* were 16,325, 16,185, 15,781, 16,101, 15,678, 16,243, 16,320, 15,825, 15,658, 15,724, 15,781, and 15,698 bp, respectively. The mitogenome map of a representative species of *Chironomus* (*Chironomus annularius*) is shown in Figure 1.

**Figure 2:**
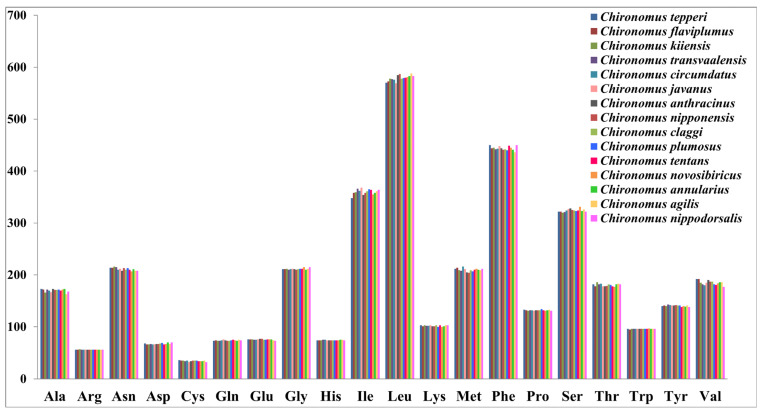
Amino acid distribution of mitogenomes of 15 *Chironomus* species. The *x* axis represents the codon families, and the *y* axis represents the total codon.

The nucleotide composition of the 15 *Chironomus* species was similar (Table S2). The complete mitogenomes of *Chironomus* were obviously inclined to A and T with the A+T content ranging from 75.31% (*Chironomus anticinus*) to 78.54% (*Chironomus transvaalensis*), a similar A+T content to other chironomids [9,10,39]. In the mitogenomes of the 15 *Chironomus* species, the A+T content of the PCGs ranged from 71.66% to 76.00%, with negative AT-skew and negative GC-skew, except in *Chironomus flaviplumus* (0.01), *Chironomus transvaalensis* (0.01), and *Chironomus circumumdatus* (0.01). In all 15 *Chironomus* species, the A+T content of the third codon of the PCGs was significantly higher than that of the first and second codons of the PCGs. Three codon positions of the PCGs all exhibited negative AT-skew. The first codon position of the PCGs exhibited a positive GC-skew while the second and third codon positions of the PCGs exhibited negative GC-skew. The start codon of the 13 PCGs was usually in the form of ATN. However, several PCGs exhibited different forms of start codon. For example, the start codon of the *COI* gene in 14 *Chironomus* species was TTG, except *Chironomus flaviplumus* (Table S3). The start codon of the *ND1* gene in 12 *Chironomus* species was TTG (Table S3). The start codon of *ND5* in all *Chironomus* species was GTG (Table S3). In the 15 *Chironomus* species, the termination codon of most PCGs was TAA, while a few genes used TAG as the termination codon or ended with an incomplete termination codon (TA-) (Table S3). The total codon length (excluding the termination codon) of the 15 *Chironomus* species ranged from 3727 to 3729 bp. Leu, Phe, and Ile were the most frequent codon families, and Cys was the least frequent codon family (Figure 2). The RSCUs of the 15 *Chironomus* species were similar.

**Figure 3:**
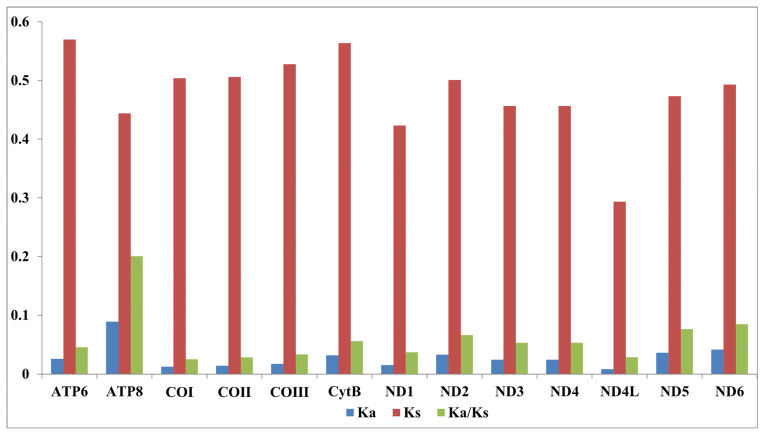
Evolution rate of 13 PCGs of mitogenomes of 15 *Chironomus* species. Ka refers to non-synonymous nucleotide substitutions, Ks refers to synonymous nucleotide substitutions, Ka/Ks refers to the selection pressure of each PCG. The *x* axis represents 13 PCGs, and the *y* axis represents the value.

The 20 amino acids were identified in all *Chironomus* species, and the common codon pattern of each amino acid was NNA or NNU. We used the Ka/Ks value (ω) to measure the extent to which species were affected by natural selection. ω of 13 PCGs are shown in Figure 3. The ω value of each PCG was less than 1, showing that the non-synonymous substitution rate was less than the synonymous substitution rate, and indicating that 13 PCGs evolved under purifying selection pressure. *ATP8* exhibited the highest ω value, while *COI* exhibited the lowest ω value, which was similar to other chironomids [8,9,10].

The 22 tRNAs were from the mitogenomes of all *Chironomus* species. The A+T content of tRNAs ranged from 78.85% to 79.88% (Table S1), exhibiting positive AT-skew and positive GC-skew. The length of 12S rRNA ranged from 813 to 821 bp, and its A+T content ranged from 82.72% to 83.99% (Table S1). The length of 16S rRNA ranged from 1336 to 1383 bp, and its A+T content ranged from 84.30% to 85.57% (Table S1). Among all *Chironomus* species, both 12S and 16S rRNA genes showed positive AT-skew and positive GC-skew, except for *Chironomus anthracinus*, *Chironomus flaviplumus*, and *Chironomus claggi* (Table S1). The size of the CR of the 15 *Chironomus* species ranged from 498 to 526 bp. The content of A+T in the CR was obviously higher than that in other regions of the mitogenome, varying from 91.29% to 95.96% and exhibiting negative AT-skew (−0.12 to −0.03) and negative GC-skew (−0.55 to −0.14).

### 3.2. Phylogenetic Analysis

**Figure 4:**
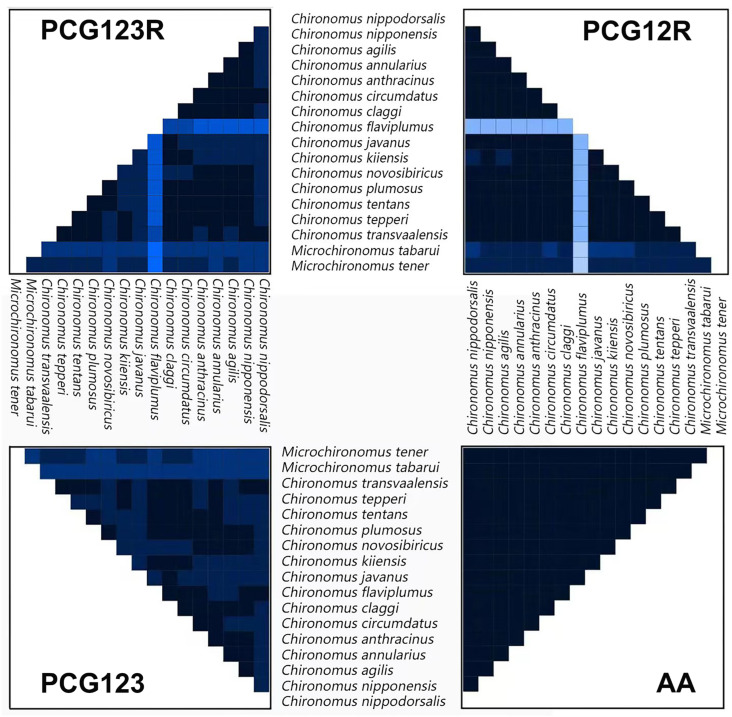
Heterogeneity analysis based on 13 PCGs and two rRNA sequences. Analysis based on AliGROOVE scores ranging from −1 (strong heterogeneity between datasets; the color is red) to +1 (weak heterogeneity between datasets; the color is blue); the lighter the color of the color block of each dataset, the stronger the heterogeneity, and the darker the color, the weaker the heterogeneity.

The results of the heterogeneity test of the four datasets (PCG123R, PCG12R, PCG123, and AA) are shown in Figure 4. The heterogeneity of the PCG12R dataset was lower than that of the PCG123 and PCG123R datasets. It could be inferred that the evolution rate of the third codon position of the PCGs was relatively high. The heterogeneity of the AA dataset was significantly reduced, indicating that even if the third codon of the PCG changed greatly, the codon was likely to be a synonymous codon encoding the same amino acid. In the PCG123R and PCG12R datasets, the heterogeneity of *Chironomus flaviplumus* was higher than that of other species, while in the PCG dataset, the heterogeneity of *Chironomus flaviplumus* was close to that of other *Chironomus* species. The reason for this might be that the rRNA sequences of *Chironomus flaviplumus* were quite different from the other 14 *Chironomus* species. The heterogeneity of two *Microchironomus* species was higher than that of other species in most datasets.

**Figure 5:**
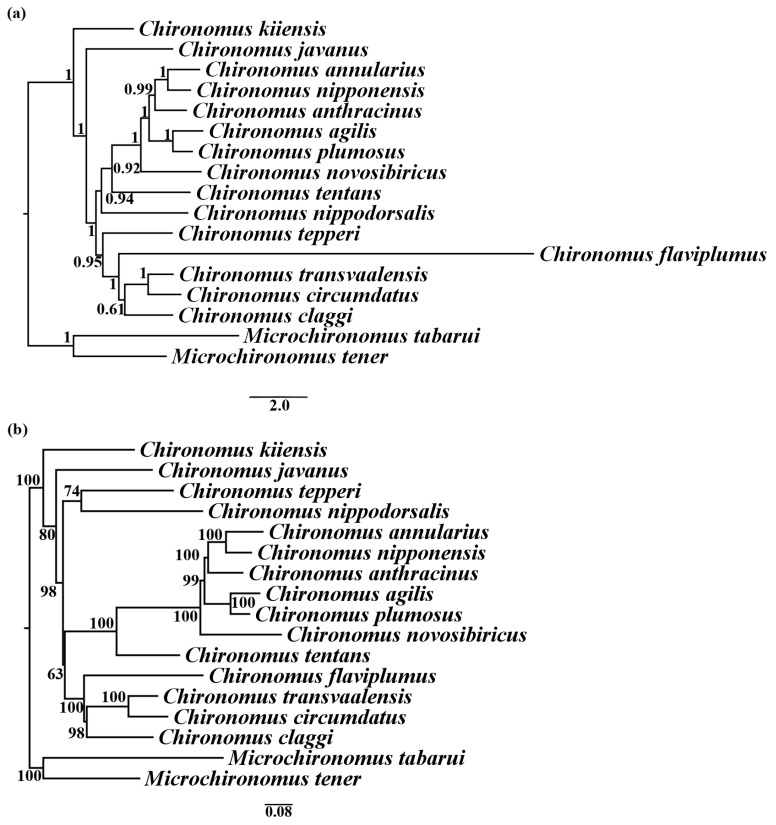
Phylogenetic trees of *Chironomus* inferred from the PCG123R dataset. (**a**) BI tree. Numbers at the nodes were BI posterior probabilities. (**b**) ML tree. Numbers at the nodes were ML bootstrap values.

In this study, the phylogenetic relationships of *Chironomus* and *Mircochironomus* were reconstructed by four datasets (PCG123R, PCG12R, PCG123, and AA). All phylogenetic trees showed that the 15 *Chironomus* species grouped in one clade, separating from *Microchironomus* (Figure 5 and Figures S2–S4). However, phylogenetic relationships among *Chironomus* were not well supported at the species level. This might be a result of the fast mutation rates in the mitogenomes of most chironomids [40]. For instance, the long branch of *C. flaviplumus* might be a result of its high mutation rate in rRNA sequences (Figure 4 and Figures S2–S4). In this study, we sampled about 1/20 of the described *Chironomus* species. Insufficient taxon sampling resulted in a lack of information on other species, and the evolutionary relationships between some species could not be highly resolved. Therefore, our phylogeny results were inconclusive for the monophyly of *Chironomus*. To explore the evolutionary history of *Chironomus*, more comprehensive taxon sampling and nuclear markers are needed.

## 4. Conclusions

This study provided the complete mitogenomes of 12 *Chironomus* species and one *Microchironomus* species for the first time and combined the public data to analyze the general features and phylogenic relationships within *Chironomus*. It showed that the nucleotide composition and gene order of the mitogenome of *Chironomus* species were conservative. Our study enriches the library of mitogenomes of chironomids and provides a valuable resource for understanding the evolutionary history of *Chironomus*.

## Figures and Tables

**Table 1:** Detailed information of 15 *Chironomus* and two *Microchironomus* species used in the study.

Species	Sample ID	Life Stage	Sampling Metadata	GenBank Accession	Reference
*Microchironomus tabarui*	XL3993	Adult male	Hengshui, Hebei, China, 37.651626°N, 115.650831°E, 1 September 2020, leg. X.-Y. Liu	MZ261913	[23]
*Microchironomus tener*	XL1462	Adult male	Changjiang, Hainan, China, 19.11463°N, 109.08419°E, 13 March 2016, leg. B.-J. Sun	ON975027	this study
*Chironomus tepperi*	JN861749	NA	NA	JN861749	[19]
*Chironomus flaviplumus*	CNUISI-020005203	Larva	Yeondeung stream, Yeosu, South Korea 34°45′26.0″ N, 127°42′51.2″ E, May 2020	MW770891	[20]
*Chironomus kiiensis*	BSZ21	Larva	Lishui, Zhejiang, China, 28°39′30′′ N, 120°5′29′′ E, August 2019, leg. X. Qi	MZ150770	[21]
*Chironomus transvaalensis*	NAM96	Larva	Goreangab Dam, Khomas, Windhoek, Namibia, 22.5267°S, 17.0153°E, 3 December 2018, leg. X.-L. Lin	ON975023	this study
*Chironomus circumdatus*	NEC119	Larva	Kouembélia, Tontouta, New Caledonia, 22.0083056°S, 166.2062775°E, 11 May 2020, leg. N. Mary	ON975024	this study
*Chironomus javanus*	NLCH300	Adult male	Yizhang, Chenzhou, Hunan, China, 24.9854183°N, 112.914357°E, 30 August 2020, leg. X.-L. Lin	ON975025	this study
*Chironomus anthracinus*	XL575	Adult male	Lian lake, Trondheim, Norway, 63.39989°N, 10.31761°E, 17 June 2016, leg. X.-L. Lin	ON975026	this study
*Chironomus nipponensis*	X2896	Larva	Laotuding, Huanren, Benxi, Liaoning, China, 41.2894°N, 124.8980°E, 3 September 2014, leg. C. Song	ON975028	this study
*Chironomus claggi*	XL2930	Larva	Maoyangzhen, Wuzhishan Hainan, China, 18.93696°N, 109.50804°E, 6 December 2010, leg. F.-Q. Kong	ON975029	this study
*Chironomus plumosus*	XL3435	Larva	Yuqiao Reservoir, Jizhou, Tianjin, China, 40.01974°N, 117.6389°E, 21 November 2019, leg. H.-J. Yu	ON975030	this study
*Chironomus tentans*	XL3813	Larva	Naqu, Xizang, China, 31.621813°N, 91.739874°E, 3 September 2020, leg. Y. Peng	ON975031	this study
*Chironomus novosibiricus*	XL3834	Larva	Zegucuo, Shannan, Xizang, China, 28.754153°N, 91.676359°E, 30 August 2020, leg. Y. Peng	ON975032	this study
*Chironomus annularius*	XL3838	Larva	Zegucuo, Shannan, Xizang, China, 28.754153°N, 91.676359°E, 30 August 2020, leg. Y. Peng	ON975033	this study
*Chironomus agilis*	XL4188	Adult male	Chun′an, Hangzhou, Zhejiang, China, 29.567168°N, 118.86825°E, 8 May 2021, leg. Y.-Y. Han	ON975034	this study
*Chironomus nippodorsalis*	XL4371	Adult female	Hefeng, Hubei, China, 29.89269645°N, 110.0287267°E, 12-July-15, leg. Q. Wang	ON975035	this study

## Data Availability

The following information was supplied regarding the availability of DNA sequences: The new mitogenomes are deposited in GenBank of NCBI under accession numbers ON975023-ON975035.

## Supplementary Materials

The following supporting information can be downloaded at: https://www.mdpi.com/article/10.3390/insects13121164/s1, Table S1: The best model for each partition of the five datasets. Table S2: Nucleotide composition of mitochondrial genomes of 15 *Chironomus* species. Table S3: Start and stop codons of 13 PCGs in the mitogenomes of 15 *Chironomus* species. Figure S1: Substitution patterns of the PCG123R (a), PCG12R (b), and PCG123 (c) datasets. The graphs represent the increase in GTR distance. Figure S2: Phylogenetic trees of *Chironomus* inferred from the AA dataset. (a) BI tree. Numbers at the nodes are BI posterior probabilities. (b) ML tree. Numbers at the nodes are ML bootstrap values. Figure S3: Phylogenetic trees of *Chironomus* inferred from the PCG123 dataset. (a) BI tree. Numbers at the nodes are BI posterior probabilities. (b) ML tree based. Numbers at the nodes are ML bootstrap values. Figure S4: Phylogenetic trees of *Chironomus* inferred from the PCG12R dataset. (a) BI tree. Numbers at the nodes are BI posterior probabilities. (b) ML tree. Numbers at the nodes are ML bootstrap values.
