# Supplementary material for: Comparative Analysis of Mitogenomes of Chironomus (Diptera: Chironomidae)
Source: Insects. 2022 Dec 16;13(12):1164. doi: 10.3390/insects13121164 (PMC9784984; doi:10.3390/insects13121164)
Supplement: Supplementary file 1 [file insects-13-01164-s001.zip › Supplementary Materials.pdf]

Supplementary materials

Table S1. The best model for each partition of the five datasets.

Datasets	Partition names	Best model
PCG123	CO3_pos1, CO1_pos1, ATP6_pos1, CytB_pos1, CO2_pos1	GTR+I+G
	CO3_pos2, CO2_pos2, CO1_pos2, ATP6_pos2	F81+I
	ATP6_pos3, ND3_pos3, CytB_pos3, CO3_pos3, ATP8_pos3, CO2_pos3	HKY+I+G
	ATP8_pos2, ATP8_pos1, ND6_pos1	HKY+I+G
	CO1_pos3, ND2_pos3, ND6_pos3	HKY+G
	ND4L_pos2, ND4L_pos1, CytB_pos2, ND1_pos2, ND3_pos2, ND4_pos2, ND6_pos2, ND5_pos2, ND2_pos2	GTR+I+G
	ND3_pos1, ND2_pos1, ND1_pos1, ND5_pos1, ND4_pos1	GTR+I+G
	ND4L_pos3, ND4_pos3, ND1_pos3, ND5_pos3	GTR+G
PCG12R	16S, 12S	HKY+G
	ATP6, CO1, CO2, CytB, CO3	GTR+I+G
	ATP8, ND2, ND6	HKY+I+G
	ND4L, ND1, ND3, ND5, ND4	GTR+I+G
PCG123R	16S, 12S	HKY+G
	CO3_pos1, CO1_pos1, ATP6_pos1, CO2_pos1, CytB_pos1	GTR+I+G
	CO2_pos2, CO3_pos2, ATP6_pos2, CO1_pos2	F81+I
	CO3_pos3, CytB_pos3, CO2_pos3, ATP8_pos3, ATP6_pos3, ND2_pos3, ND6_pos3	HKY+I+G
	ATP8_pos2, ATP8_pos1, ND6_pos1	HKY+I+G
	CO1_pos3, ND3_pos3	HKY+G
	ND4L_pos1, ND4L_pos2, CytB_pos2, ND3_pos2, ND1_pos2, ND4_pos2, ND6_pos2, ND2_pos2, ND5_pos2	GTR+I+G
	ND1_pos1, ND5_pos1, ND4_pos1, ND2_pos1, ND3_pos1	GTR+I+G
	ND4L_pos3, ND1_pos3, ND4_pos3, ND5_pos3	GTR+G
AA	CO1, CO2, ND4L, ATP6	MTMAM+I+G
	ATP8, ND6	MTREV+I+G
	CO3, ND2, ND3, ND4, CytB, ND1	MTMAM+I+G
	ND5	MTMAM+I+G

Table S2. Nucleotide composition of mitochondrial genomes of 15 *Chironomus* species.

	Species	Whole Genome	Protein Coding Genes	1st Codon Position	2nd Codon Position	3rd Codon Position	tRNA Genes	12S rRNA	16S rRNA	Control Region
A+T %	*Chironomus anthracinus*	75.31	71.66	66.81	67.69	80.46	78.85	82.89	84.35	91.29
	Chironomus nipponensis	76.13	72.68	67.93	67.80	82.31	79.13	82.95	84.79	92.11
	Chironomus flaviplumus	78.47	76.00	69.13	67.72	91.15	79.21	83.25	84.88	95.38
	Chironomus plumosus	76.20	72.93	67.74	67.66	83.38	79.43	83.99	84.89	92.81
	Chironomus tentans	76.94	74.06	68.17	67.85	86.16	79.44	82.82	85.24	93.57
	Chironomus novosibiricus	76.19	72.72	67.58	67.56	83.03	79.13	82.80	85.18	93.36
	Chironomus annularius	75.56	72.33	67.91	67.75	81.33	79.10	83.07	84.70	91.90
	Chironomus agilis	75.64	72.34	67.44	67.79	81.80	79.17	82.97	85.01	92.40
	Chironomus nippodorsalis	76.40	73.40	68.12	67.85	84.23	79.16	83.54	84.73	92.32
	Chironomus tepperi	76.92	74.33	67.75	67.67	87.58	79.02	82.60	84.30	93.00
	Chironomus claggi	77.83	75.26	68.76	67.88	89.15	79.08	82.74	84.52	94.29
	Chironomus kiiensis	76.94	74.21	71.88	67.99	82.75	79.23	82.72	84.66	90.87
	Chironomus transvaalensis	78.54	75.92	69.24	67.82	90.70	79.88	83.42	85.57	95.96
	Chironomus circumdatus	78.44	75.87	69.27	67.72	90.62	79.71	83.15	85.15	94.75
	Chironomus javanus	77.37	74.57	68.04	67.93	87.73	79.59	82.97	85.03	95.15
AT-skew	*Chironomus anthracinus*	0.03	-0.20	-0.08	-0.40	-0.14	0.03	-0.01	0.00	-0.09
	Chironomus nipponensis	0.03	-0.20	-0.09	-0.40	-0.13	0.04	0.01	0.01	-0.10
	Chironomus flaviplumus	0.01	-0.20	-0.10	-0.40	-0.11	0.02	0.00	0.03	-0.03
	Chironomus plumosus	0.03	-0.20	-0.08	-0.40	-0.13	0.03	0.02	0.00	-0.06

	Chironomus tentans	0.02	-0.19	-0.09	-0.40	-0.10	0.04	0.01	0.02	-0.06
	Chironomus novosibiricus	0.03	-0.20	-0.09	-0.40	-0.13	0.05	0.01	0.01	-0.07
	Chironomus annularius	0.03	-0.20	-0.09	-0.40	-0.12	0.03	0.02	0.01	-0.09
	Chironomus agilis	0.03	-0.20	-0.08	-0.40	-0.12	0.04	0.01	0.01	-0.10
	Chironomus nippodorsalis	0.02	-0.19	-0.09	-0.41	-0.10	0.04	0.02	0.00	-0.06
	Chironomus tepperi	0.02	-0.19	-0.09	-0.40	-0.12	0.04	0.01	0.00	-0.10
	Chironomus claggi	0.01	-0.19	-0.10	-0.40	-0.11	0.03	-0.01	-0.01	-0.10
	Chironomus kiiensis	0.02	-0.18	-0.08	-0.36	-0.13	0.04	0.01	-0.02	-0.10
	Chironomus transvaalensis	0.01	-0.19	-0.10	-0.40	-0.11	0.04	0.00	0.01	-0.12
	Chironomus circumdatus	0.01	-0.20	-0.10	-0.40	-0.13	0.04	0.00	0.01	-0.07
	Chironomus javanus	0.01	-0.20	-0.08	-0.40	-0.14	0.03	0.01	0.00	-0.11
GC-skew	*Chironomus anthracinus*	-0.20	-0.04	0.15	-0.17	-0.18	0.13	0.36	0.39	-0.23
	Chironomus nipponensis	-0.21	-0.02	0.19	-0.18	-0.13	0.12	0.37	0.37	-0.35
	Chironomus flaviplumus	-0.17	0.01	0.23	-0.18	-0.03	0.15	0.36	0.36	-0.25
	Chironomus plumosus	-0.23	-0.04	0.17	-0.18	-0.18	0.14	0.33	0.39	-0.55
	Chironomus tentans	-0.21	-0.04	0.18	-0.18	-0.22	0.12	0.36	0.38	-0.50
	Chironomus novosibiricus	-0.19	-0.03	0.18	-0.17	-0.15	0.11	0.35	0.36	-0.29
	Chironomus annularius	-0.22	-0.02	0.19	-0.18	-0.10	0.13	0.35	0.39	-0.32
	Chironomus agilis	-0.22	-0.05	0.15	-0.18	-0.18	0.12	0.37	0.39	-0.33
	Chironomus nippodorsalis	-0.20	-0.03	0.18	-0.18	-0.13	0.13	0.34	0.37	-0.15
	Chironomus tepperi	-0.18	-0.01	0.19	-0.18	-0.12	0.14	0.37	0.36	-0.43
	Chironomus claggi	-0.18	-0.01	0.22	-0.18	-0.13	0.06	-0.36	-0.35	-0.31

	Chironomus kiiensis	-0.17	-0.02	0.15	-0.10	-0.13	0.13	0.35	0.35	-0.25
	Chironomus transvaalensis	-0.17	0.01	0.22	-0.18	-0.03	0.15	0.35	0.36	-0.14
	Chironomus circumdatus	-0.18	0.01	0.22	-0.18	-0.03	0.14	0.36	0.38	-0.41
	Chironomus javanus	-0.19	-0.01	0.18	-0.17	-0.08	0.15	0.37	0.34	-0.36

Table S3 Start and stop codons of 13 PCGs in the mitogenomes of 15 *Chironomus* species.

Feature	Start/Stop codon												
	ATP8	*ATP6*	*CO1*	*CO2*	*CO3*	*CytB*	*ND1*	*ND2*	*ND3*	*ND4*	*ND4L*	*ND5*	*ND6*
Chironomus tepperi	ATC/TAA	ATG/TAA	TTG/TAG	ATG/TAA	ATG/TAA	ATG/TAA	ATT/TAA	ATT/TAA	ATT/TAA	ATG/TAA	ATG/TAA	GTG/TAA	ATT/TAA
Chironomus flaviplumus	ATT/TAA	ATG/TAA	ATA/TAA	ATG/TAA	ATG/TAA	ATG/TAG	ATT/TAA	ATT/TAA	ATT/TAA	ATG/TAA	ATG/TAA	GTG/TAA	ATT/TAA
Chironomus kiiensis	ATC/TAA	ATG/TAA	TTG/TAA	ATG/TAA	ATG/TAA	ATG/TAA	ATT/TAA	ATT/TAA	ATT/TAA	ATG/TAG	ATG/TAA	GTG/TAA	ATT/TAA
Chironomus transvaalensis	ATT/TAA	ATG/TAA	TTG/TAA	ATG/TAA	ATG/TAA	ATG/TAA	TTG/TAA	ATT/TAA	ATT/TAA	ATG/TAA	ATG/TAA	GTG/TAA	ATT/TAA
Chironomus circumdatus	ATC/TAA	ATG/TAA	TTG/TAA	ATG/TAA	ATG/TAA	ATG/TAA	TTG/TAA	ATT/TAA	ATT/TAA	ATG/TAA	ATG/TAA	GTG/TAA	ATT/TAA
Chironomus javanus	ATT/TAA	ATG/TAA	TTG/TAA	ATG/TAA	ATG/TAA	ATG/TAA	TTG/TAA	ATT/TAA	ATG/TAA	ATG/TAG	ATG/TAA	GTG/TAA	ATT/TAA
Chironomus anthracinus	ATC/TAA	ATG/TAA	TTG/TAA	ATG/TAA	ATG/TAA	ATG/TAA	TTG/TAA	ATT/TAA	ATT/TAA	ATG/TAG	ATG/TAA	GTG/TAA	ATT/TAA
Chironomus nipponensis	ATT/TAA	ATG/TAA	TTG/TAA	ATG/TAA	ATG/TAA	ATG/TAA	TTG/TAA	ATT/TA-	ATT/TAA	ATG/TAG	ATG/TAA	GTG/TAA	ATT/TAA
Chironomus claggi	ATT/TAA	ATG/TAA	TTG/TAA	ATG/TAA	ATG/TAA	ATG/TAA	TTG/TAA	ATT/TAA	ATT/TAA	ATG/TAA	ATG/TAA	GTG/TAA	ATT/TAA
Chironomus plumosus	ATT/TAA	ATG/TAA	TTG/TAA	ATG/TAA	ATG/TAA	ATG/TAA	TTG/TAA	ATT/TAA	ATT/TAA	ATG/TAA	ATG/TAA	GTG/TAA	ATT/TAA
Chironomus tentans	ATT/TAA	ATG/TAA	TTG/TAA	ATG/TAA	ATG/TAA	ATG/TAA	TTG/TAA	ATT/TAA	ATT/TAA	ATG/TAA	ATG/TAA	GTG/TAA	ATT/TAA
Chironomus novosibiricus	ATC/TAA	ATG/TAA	TTG/TAA	ATG/TAA	ATG/TAG	ATG/TAA	TTG/TAA	ATT/TAA	ATT/TAA	ATG/TAA	ATG/TAA	GTG/TAA	ATT/TAA
Chironomus annularius	ATC/TAA	ATG/TAA	TTG/TAA	ATG/TAA	ATG/TAA	ATG/TAA	TTG/TAA	ATT/TAA	ATT/TAA	ATG/TAA	ATG/TAA	GTG/TAA	ATT/TAA

Chironomus agilis	ATT/ TAA	ATG/ TAA	TTG/ TAA	ATG/ TAA	ATG/ TAA	ATG/ TAA	TTG/ TAA	ATT/ TAA	ATT/ TAA	ATG/ TAA	ATG/ TAA	GTG/ TAA	ATT/ TAA
Chironomus nippodorsalis	ATT/ TAA	ATG/ TAA	TTG/ TAA	ATG/ TAA	ATG/ TAA	ATG/ TAA	TTG/ TAA	ATT/ TAA	ATT/ TAA	ATG/ TAA	ATG/ TAA	GTG/ TAA	ATT/ TAA

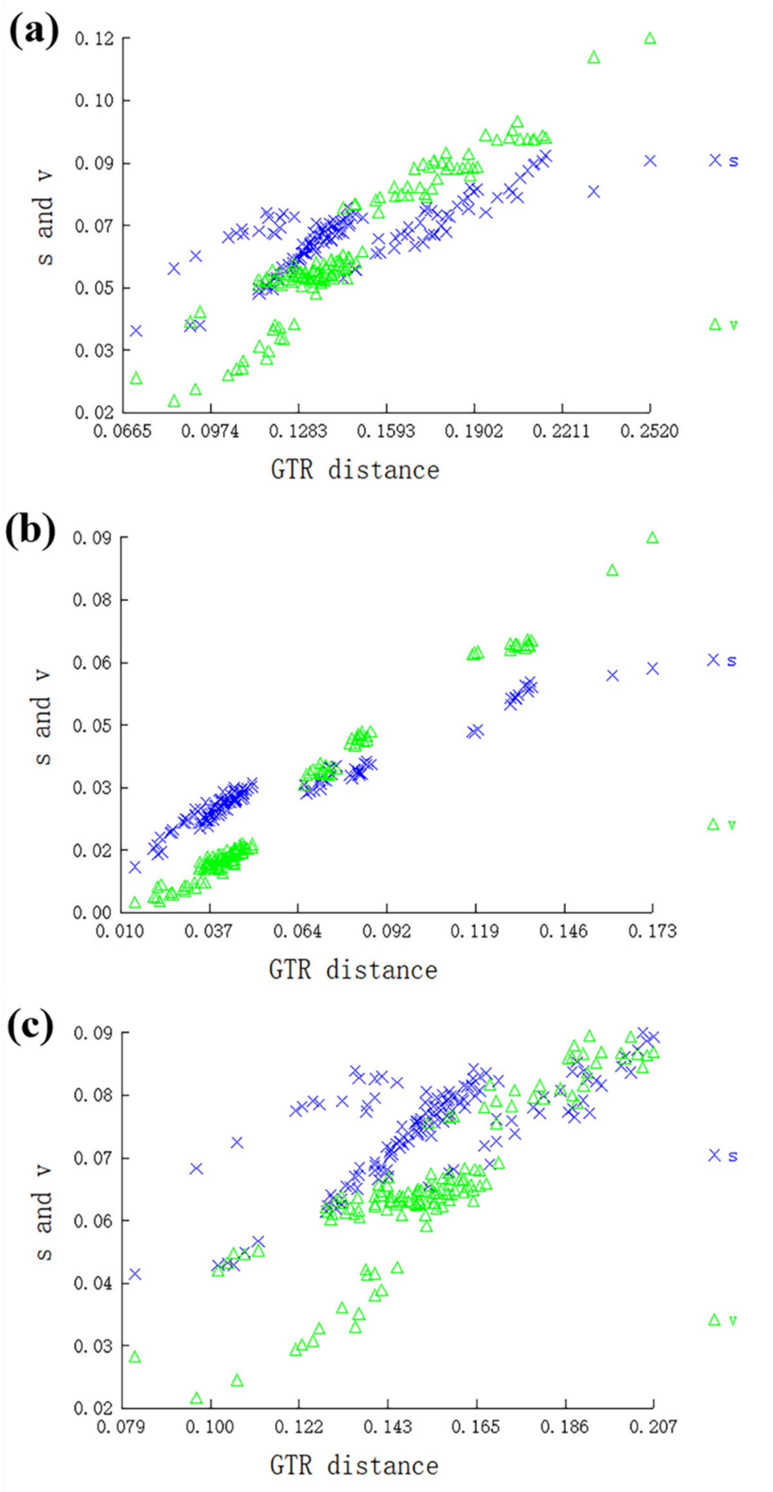

Figure S1. Substitution patterns of the PCG123R (a), PCG12R (b) and PCG123 (c) datasets. The graphs represent the increase in GTR distance.

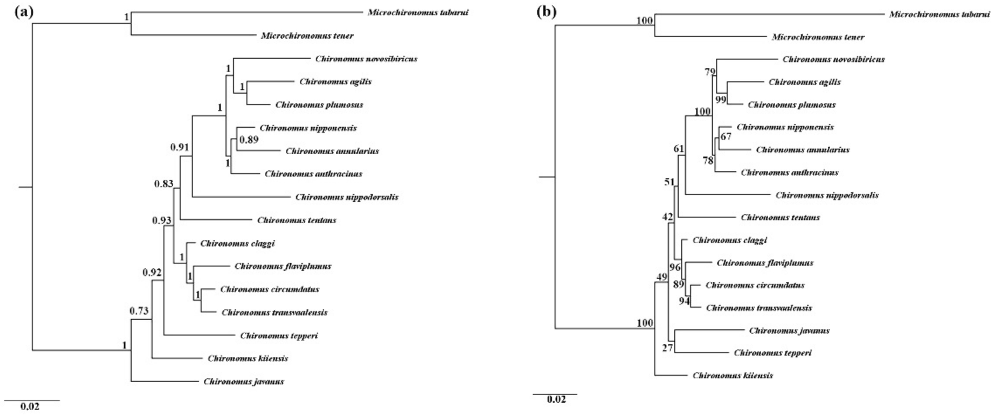

Figure S2. Phylogenetic trees of *Chironomus* inferred from the AA dataset. (a) BI tree. Numbers at the nodes are BI posterior probabilities. (b) ML tree. Numbers at the nodes are ML bootstrap values.

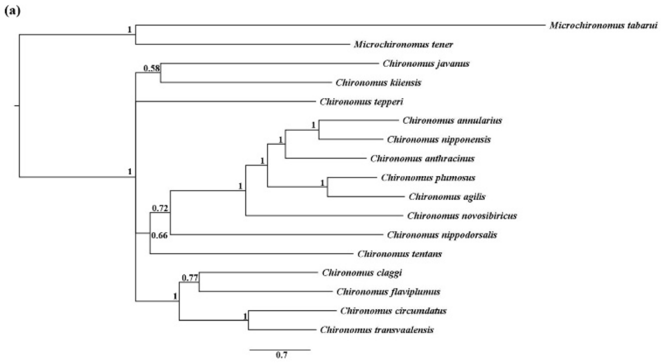

Figure S3. Phylogenetic trees of *Chironomus* inferred from the PCG123 dataset. (a) BI tree. Numbers at the nodes are BI posterior probabilities. (b) ML tree. Numbers at the nodes are ML bootstrap values.

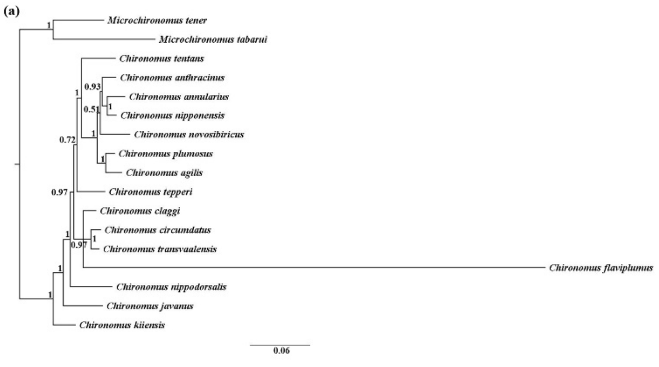

Figure S4. Phylogenetic trees of *Chironomus* inferred from the PCG12R dataset. (a) BI tree. Numbers at the nodes are BI posterior probabilities. (b) ML tree. Numbers at the nodes are ML bootstrap values.
